# Membrane Attack Complex C5b-9 Promotes Renal Tubular Epithelial Cell Pyroptosis in Trichloroethylene-Sensitized Mice

**DOI:** 10.3389/fphar.2022.877988

**Published:** 2022-05-17

**Authors:** Feng Wang, Meng Huang, Yican Wang, Yiting Hong, Dandan Zang, Chunjun Yang, Changhao Wu, Qixing Zhu

**Affiliations:** ^1^ Department of Dermatology, The Second Hospital of Anhui Medical University, Hefei, China; ^2^ Department of Occupational Health and Environmental Health, School of Public Health, Anhui Medical University, Hefei, China; ^3^ Center for Scientific Research and Experiment, Anhui Medical University, Hefei, China; ^4^ Faculty of Health and Medical Sciences, University of Surrey, Guildford, United Kingdom; ^5^ Department of Dermatology, The First Affiliated Hospital of Anhui Medical University, Hefei, China; ^6^ Key Laboratory of Dermatology, Ministry of Education, The First Affiliated Hospital of Anhui Medical University, Hefei, China

**Keywords:** trichloroethylene, membrane attack complex, renal tubular epithelial cell, pyroptosis, NLRP3 inflammasome

## Abstract

Trichloroethylene (TCE), a commonly used organic solvent, is known to cause trichloroethylene hypersensitivity syndrome (THS), also called occupational medicamentosa–like dermatitis due to TCE (OMDT) in China. OMDT patients presented with severe inflammatory kidney damage, and we have previously shown that the renal damage is related to the terminal complement complex C5b-9. Here, we sought to determine whether C5b-9 participated in TCE-induced immune kidney injury by promoting pyroptosis, a new form of programed cell death linked to inflammatory response, with underlying molecular mechanisms involving the NLRP3 inflammasome. A BALB/c mouse-based model of OMDT was established by dermal TCE sensitization in the presence or absence of C5b-9 inhibitor (sCD59-Cys, 25μg/mouse) and NLRP3 antagonist (MCC950, 10 mg/kg). Kidney histopathology, renal function, expression of inflammatory mediators and the pyroptosis executive protein gasdermin D (GSDMD), and the activation of pyroptosis canonical NLRP3/caspase-1 pathway were examined in the mouse model. Renal tubular damage was observed in TCE-sensitized mice. GSDMD was mainly expressed on renal tubular epithelial cells (RTECs). The caspase-1–dependent canonical pathway of pyroptosis was activated in TCE-induced renal damage. Pharmacological inhibition of C5b-9 could restrain the caspase-1–dependent canonical pathway and rescued the renal tubular damage. Taken together, our results demonstrated that complement C5b-9 plays a central role in TCE-induced immune kidney damage, and the underlying mechanisms involve NLRP3-mediated pyroptosis.

## Introduction

Trichloroethylene (TCE) is a chemical agent widely used for industrial degreasing and cleaning (Harris et al., 2018; Elkin et al., 2021). However, the extensive application of TCE has led to multiple health problems, especially inflammatory immune diseases such as TCE hypersensitivity syndrome (THS) or occupational medicamentosa–like dermatitis due to trichloroethylene (OMDT) in China, lupus nephritis, hepatitis, scleroderma, vasculitis, and so on (Ordaz et al., 2017a). In the past decades, occupational exposure to TCE has been reported to cause severe OMDT, manifesting as extensive skin necrosis and exfoliation similar to severe cutaneous adverse drug reactions and multiple organ injuries, including liver and kidney damage (Ordaz et al., 2017b).

The kidney damage in OMDT is immune-mediated and characterized by a tubular inflammatory reaction and the dysfunction of renal protein uptake (Liu, 2009; Wang F. et al., 2019). Animal studies have found that innate immunity, including local complement activation, is involved in the tubular damage of TCE-sensitized mice, as evidenced by increased deposition of the terminal complement activation product C5b-9 (Wang G. et al., 2020; Xie et al., 2021). C5b-9 displays both lytic and sub-lytic effects. C5b-9 can form a transmembrane channel in the cell membrane and induce target cell lysis by changing the osmotic pressure (Serna et al., 2016). On the other hand, in response to various regulatory factors, C5b-9 also exerts a sub-lytic effect (causing less than 5% death cells), which promotes the release of proinflammatory cytokines, including interleukin (IL)-1β and IL-18 (Morgan et al., 2016). Our recent studies have demonstrated that the sub-lytic C5b-9 is mainly deposited on renal tubular epithelial cells (RTECs) and that the exogenous supplement of CD59, a C5b-9 inhibitory protein, alleviates tubular damage in TCE-sensitized mice (Wang X. et al., 2019).

Pyroptosis is a newly discovered form of programed cell death induced by inflammatory caspases, including caspase-1, -4, -5, or -11 (Barnett and Ting, 2020). These caspases cleave executive protein gasdermin D (GSDMD) to form cell membrane pores, leading to the release of inflammatory mediators (Broz et al., 2020). The pyroptosis pathway was divided into the caspase-1–dependent canonical pathway and caspase-4–, caspase-5–, and caspase-11–dependent non-canonical pathways (Cheng et al., 2017). In the canonical pathway, the activation of NLRP3 triggered by endogenous or exogenous danger signals converts pro-caspase-1 into catalytically active caspase-1 and consequently mediates the maturation and release of IL-1β and IL-18 to amplify the inflammatory response (Ding et al., 2016). In the non-canonical pathway, caspase-4, -5, -11 (homologs of caspase-4 and -5 in mice) are able to directly activate GSDMD to induce pyroptosis (Kayagaki et al., 2015). Consistently, our previous study also found the activation of NLRP3 signaling in TCE-induced kidney damage in mice, which was associated with renal C5b-9 deposition (Xie et al., 2021).

Here, we questioned whether C5b-9 contributes to TCE-induced tubular inflammatory injuries by regulating RTEC pyroptosis. We hypothesized that C5b-9 activates NLRP3 signaling and promotes a caspase-1–mediated canonical pathway to trigger pyroptosis. To address this, a mouse model of OMDT through TCE sensitization was established, and renal function, kidney histopathology, expression of inflammatory mediators, and the activation of RTEC pyroptosis were examined in these mice pretreated with or without sCD59-Cys (C5b-9 inhibitor) and MCC950 (NLRP3 antagonist).

## Materials and Methods

### Antibodies and Reagents

TCE, Freund’s complete adjuvant (FCA), and collagenase A were purchased from Sigma Chemical (St. Louis, MO, United States). Olive oil and acetone were purchased from Shanghai Chemical Reagent Co., Ltd. (Shanghai, China). Soluble recombinant rat CD59-Cys (sCD59-Cys) and MCC950 were purchased from Elabscience Biotechnology Co., Ltd. (Wuhan, China) and Selleck Chemicals (Houston, TX, United States), respectively. Kits for the detection of blood urea nitrogen (BUN) and creatinine (Cre) were obtained from Nanjing Jiancheng Bioengineering Institute (Nanjing, China). Kits for the determination of α1 microglobulin (α1-MG) and β2 microglobulin (β2-MG) were acquired from Elabscience Biotechnology Co., Ltd. (Wuhan, China).

Antibodies were obtained from the following companies: rabbit monoclonal anti-cleaved GSDMD antibody (ab255603), rabbit monoclonal anti-NLRP3 antibody (ab270449), rabbit monoclonal anti-IL-18 antibody (ab223293), goat anti-mouse IgG H&L (Alexa Fluor^®^ 594) (ab150116), and goat anti-rabbit IgG H&L (Alexa Fluor^®^ 488) (ab150077) (Abcam, Cambridge, United Kingdom); rabbit polyclonal anti-GSDMD antibody (af4012), rabbit polyclonal anti-GSDME antibody (df9705), and rabbit polyclonal anti-caspase-1 and anti-caspase-1 p20 antibodies (af5418, af4005) (Affinity, United States); mice monoclonal anti-C5b-9 antibody (sc-66190), mice monoclonal anti-ASC antibody (sc-514414), and mice monoclonal anti-IL-1β antibody (sc-514414) (Santa Cruz, United States); goat anti-rabbit IgG antibody and goat anti-mouse IgG antibody (Elabscience, Wuhan, China).

### Ethics and Mice Treatments

A total of 102 female BALB/c mice (initial weight 18∼22g, 6∼8 weeks old) were obtained from the Experimental Animal Center of Anhui Medical University (Hefei, China). The mice were housed with food and water *ad libitum* under a pathogen-free condition with a 12-h light/dark cycle at 20∼25°C and 50 ± 5% relative humidity. The care and use of mice complied with the National Institutes of Health guide for the care and use of laboratory animals (NIH Publications No. 8023, revised 1978). All experimental procedures were reviewed and approved by the Experimental Animal Ethics Committee of Anhui Medical University (NO. LLSC20180310).

**FIGURE 1:**
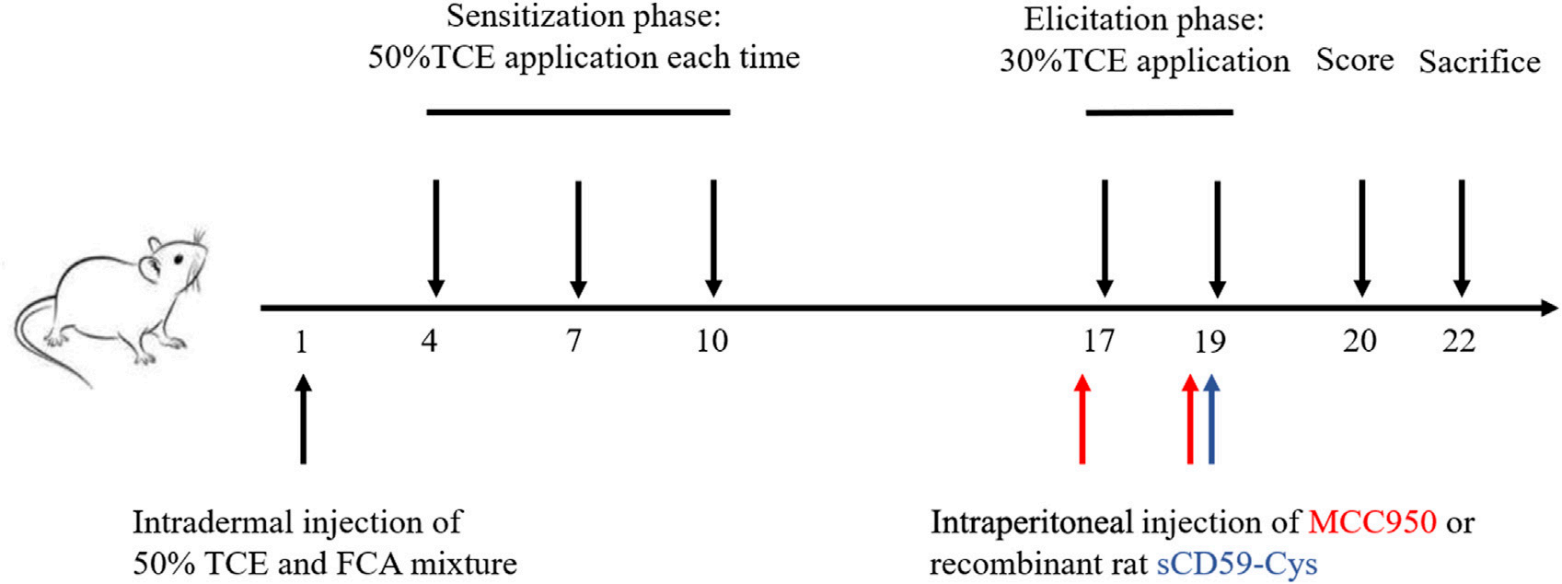
Flow diagram of the skin-sensitized mouse model by TCE. Note: 50% TCE (TCE: olive oil: acetone = 5:2:3); 30% TCE (TCE: olive oil: acetone = 3:2:5). sCD59-cys, a specific inhibitor of C5b-9 assembly; MCC950, a specific antagonist of NLRP3.

The mouse model of TCE hypersensitivity was established as described previously (Figure 1) (Wang et al., 2015). Briefly, a total mixture of 100 μL 50% TCE (TCE: acetone: olive oil = 5:3:2, v/v/v) and an equal volume of FCA was intradermally injected into the naked back skin on day 1. Then, 100 μL of 50% TCE was externally used on days 4, 7, and 10. On days 17 and 19, 100 μL of 30% TCE (TCE: acetone: olive oil = 3:5:2, v/v/v) was dropped on the dorsal skin. To block the assembly of C5b-9, sCD59-cys (25 μg/mouse) was additionally applied *via* intraperitoneal injection on day 19 (Wang F. et al., 2019). To inhibit the NLRP3 pathway, MCC950 (10 mg/kg) was additionally applied *via* intraperitoneal injection on days 17 and 19. On day 20, the cutaneous reactions were scored on a 4-point scale (no reaction, 0-point; scattered mild redness, 1-point; moderate and diffuse redness, 2-points; intensive erythema and swelling, 3-points). A total score≥1 was defined as positive sensitization, and a score = 0 was set as negative sensitization. The mice receiving saline only were set as the blank control. The mice receiving the same dose of acetone and olive oil without TCE were determined as the vehicle control. According to the cutaneous reaction score and subsequent treatment, the mice were divided into the TCE sensitization–positive group (TCEpos), TCE sensitization–negative group (TCEneg), sCD59-cys pretreatment + TCE sensitization–positive group (CD59 + TCEpos), sCD59-cys pretreatment + TCE sensitization–negative group (CD59 + TCEneg), MCC950 pretreatment + TCE sensitization–positive group (MCC950 + TCEpos), and MCC950 pretreatment + TCE sensitization–negative group (MCC950 + TCEneg).

### Renal Function Test

Mouse blood was collected from the ocular venous plexus after being anesthetized by CO_2_ inhalation on day 22. The levels of BUN, Cre, α1-MG, and β2-MG were measured to assess the renal function according to the manufacturer’s instructions of the assay kit.

### Histopathology of Renal Tubules

Hematoxylin & Eosin (H&E) staining and transmission electron microscope observation were performed to assess the pathological damage of renal tubules and ultrastructural damage of RTECs. The procedures were consistent with those of our previous studies. After proper staining, the sections were scanned under an Olympus BX53 optical microscope (Tokyo, Japan) or a JEM-1230 transmission electron microscope (Tokyo, Japan).

### Immunohistochemistry and Immunofluorescence

For the immunohistochemistry test, the paraffin-embedded cortex section was dewaxed and rehydrated with xylene and gradient ethanol, permeabilized in 0.1% Triton X-100 for 30 min, and endogenous peroxidase was blocked with 3% H_2_O_2_. The sections were then heated by a microwave oven to retrieve antigens in citrate solution and treated with goat serum to block nonspecific antigens. The primary antibodies to C5b-9, NLRP3, GSDMD, IL-1β, and IL-18 were applied overnight at 4°C. The next day, the sections were washed with PBS and treated with goat anti-rabbit or goat anti-mouse IgG at 37°C for 30 min. Then, a horseradish enzyme–labeled streptavidin working solution was applied, and the DAB kit (ZSGB-BIO, Beijing, China) was used to stain the sections according to the manufacturer’s instructions.

For the immunofluorescence test, the cortex sections were incubated with anti–cytokeratin18 and anti-GSDMD at 4°C overnight after dewaxing, hydration, permeability, antigen repairment, and blocking. Then, the sections were incubated with fluorescent secondary antibodies at room temperature for 2 h. DAPI solution (Solarbio, Beijing, China) was used to stain the cell nucleus, and the sections were scanned and photographed under an inverted fluorescence microscope or an Olympus IX73 optical microscope (Tokyo, Japan).

### RTEC Isolation

RTECs were harvested from the renal cortex of mice following a classical protocol published previously^18^. The minced cortex was digested with the appropriate volume of DMEM/F12 medium containing 0.1% collagenase I at 37°C for 30 min. Then, an equal volume of pre-chilled HBSS was added to terminate digestion. Subsequently, a series of cell sieves (including 250 and 75 μm mesh diameters) were used to collect RTECs.

### Western Blot Assay

The total proteins were extracted using radio immuno-precipitation lysis buffer (RIPA) containing PMSF and phosphatase inhibitor. Protein concentrations were quantified *via* the BCA Protein Assay Kit (Beyotime Institute of Biotechnology, China). For immunoblots, the same amount of protein (50 μg) was separated electrophoretically by SDS-PAGE and transferred to an enhanced nitrocellulose membrane. After blocking in 5% non-fat powdered milk for 2 h at room temperature, the membrane was incubated with the primary antibodies overnight at 4°C as follows: GSDMD (diluted at 1:1,000), cleaved-GSDMD (diluted at 1:1,000), NLRP3 (diluted at 1:1,000), ASC (diluted at 1:1,000), caspase-1 (diluted at 1:1,000), caspase-1 p20 (diluted at 1:1,000), and GAPDH (diluted at 1:10,000). Then, the membrane was incubated with goat anti-rabbit IgG (diluted at 1:5,000) or goat anti-mouse IgG (diluted at 1:5,000) for 2 h. After incubation with the SuperSignal West Femto Maximum Sensitivity Substrate, the membrane was detected in a chemiluminescence system.

### Quantitative Real-Time PCR Assay

Total RNA was extracted by TRIzol reagent, and the concentration and purity were determined by NanoDrop One. The initial concentration was diluted to 500 ng/μL with RNase-free water. The cDNA was obtained by the RevertAid First-Strand cDNA Synthesis Kit (Thermo Scientific, MA, United States) according to the manufacturer’s manual. Then, PCR amplification was carried out with an SYBR Green I Master kit in a Light Cycler 480 system (Roche, Switzerland). The amplification program was set as follows: initial denaturation, 95°C for 15min; denaturation, 95°C for 15 s, 45 cycles; annealing, 55°C for 15 s; extension, 72°C for 30 s. The primer sequences are listed in Table 1.

**TABLE 1:** Primers for RT-PCR used in the present study.

Gene names	Sequences (5ʹ–3ʹ)
GSDMD	Forward: 5′-CTA​GCT​AAG​GCT​CTG​GAG​ACA​A
	Reverse: 5′-GAT​TCT​TTT​CAT​CCC​AGC​AGT​C-3′
GSDME	Forward: 5′-GAG​AGT​CAC​TCT​TCG​TTT​GGA​A-3′
	Reverse: 5′-CTG​AAG​TAC​CAG​GTT​GTC​CAT​A-3′
Caspase-1	Forward: 5′-AGA​GGA​TTT​CTT​AAC​GGA​TGC​A-3′
	Reverse: 5′-TCA​CAA​GAC​CAG​GCA​TAT​TCT​T-3′
Caspase-11	Forward: 5′-TGC​AGA​GCT​ATT​ACT​CGC​GG-3′
	Reverse: 5′-ACA​GGC​AGC​TGA​GAA​CCA​TC-3′
GAPDH	Forward: 5′-CCC​TTA​AGA​GGG​ATG​CTG​CC-3′
	Reverse: 5′-ACT​GTG​CCG​TTG​AAT​TTG​CC-3′

GSDMD, gasdermin D; GSDME, gasdermin E.

### Statistical Analysis

All data were expressed as mean ± standard deviation (SD) and performed with SPSS 23.0. The *Kolmogorov–Smirnov* test was evaluated for the normal distribution. The *chi-square* test was used to examine the difference in sensitization rates in various groups. *One-way analysis of variance (ANOVA)* followed by *Tukey’s* test was used to determine differences between multiple groups. A *p* value < 0.05 was considered statistically significant.

## Results

### Renal Tubular Inflammatory Damage was Present in TCE-Sensitized Mice

**FIGURE 2:**
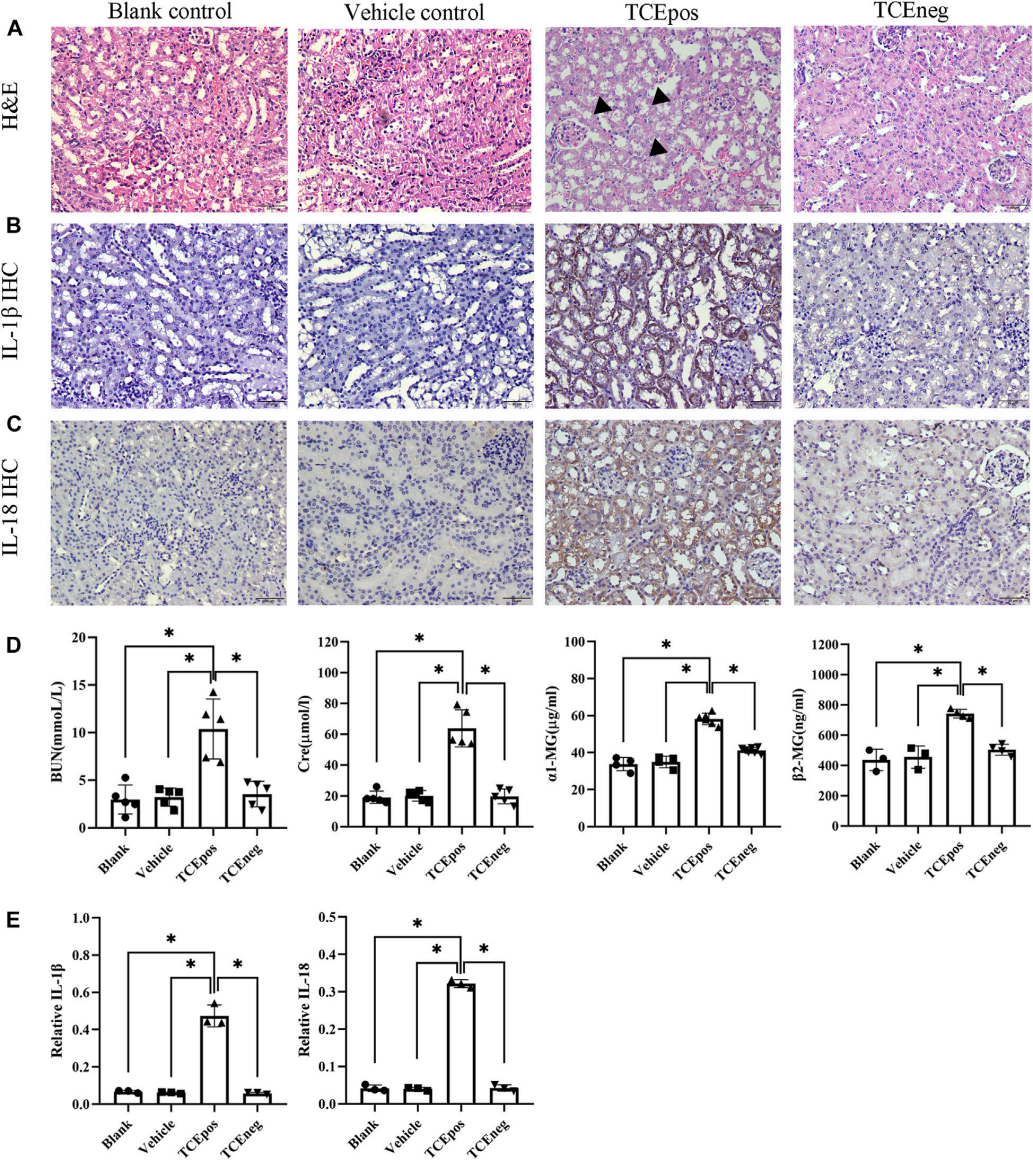
Renal tubular inflammatory damage was present in TCE-sensitized mice. **(A)** Hematoxylin & Eosin (H&E) staining of kidney and **(B,C)** immunohistochemistry (IHC) of IL-1β and IL-18 in mice. The black arrow shows tubular swelling, vacuolar degeneration, or detachment of renal tubular epithelial cells. Magnification, ×400. The scale bars represent 50 μm. **(D)** Measurement of renal function including the levels of blood urea nitrogen (BUN), creatinine (Cre), α1 micro-globulin (α1-MG), and β2 micro-globulin (β2-MG). **(E)** Relative expression of IL-1β and IL-18 was calculated by average optical density in mice. Data are representative of three independent experiments and are expressed as the mean ± SD. *n* = 3–5/group. Levels of significance were defined as below: *p < 0.05.

The mouse groups and total sensitization rate are listed in Table 2. H&E staining showed intact structures of the renal tubules with an orderly arrangement of kidney cells in the blank control group, vehicle control group, and TCEneg group. However, tubule dilatation with RTECs and renal interstitial edema was present in the TCEpos group (Figure 2A). The immunohistochemistry test showed an increased expression of inflammatory cytokines IL-1β and IL-18 in the TCEpos group (Figures 2B,C), as compared to the TCEneg group (*p* < 0.05). No statistical differences of IL-1β and IL-18 were found among the blank control group, vehicle control group, and TCEneg group (*p* > 0.05) (Figure 2E). Furthermore, the renal tubular function was further assessed by the detection of serum BUN, Cre, α1-MG, and β2-MG. Compared to the vehicle control group, the serum levels of BUN, Cre, α1-MG, and β2-MG were upregulated in the TCEpos group (*p* < 0.05), while no significant differences were found among the blank control group, vehicle control group, and TCEneg group (*p* > 0.05) (Figure 2D).

**TABLE 2:** Groups and sensitization rate of mice in the present study.

Groups	Mice	Score				Sensitization rate (%)
	n	0	1	2	3	
Blank control	5	5	0	0	0	0.00
Vehicle control	5	5	0	0	0	0.00
TCE treatment	30	22	7	1	0	26.67
TCEpos	8	0	7	1	0	—
TCEneg	22	22	0	0	0	—
sCD59-cys + TCE treatment	26	19	5	2	0	26.92
CD59 + TCEpos	7	0	5	2	0	—
CD59 + TCEneg	19	19	0	0	0	—
MCC950 + TCE treatment	36	23	9	4	0	36.11
MCC950 + TCEpos	13	0	9	4	0	—
MCC950 + TCEneg	23	23	0	0	0	—

TCE, trichloroethylene; pos, positive; neg, negative; sCD59-cys, a specific inhibitor of C5b-9 assembly; MCC950, a specific antagonist of NLRP3.

### GSDMD-Mediated RTEC Pyroptosis Contributed to TCE-Induced Renal Damage

**FIGURE 3:**
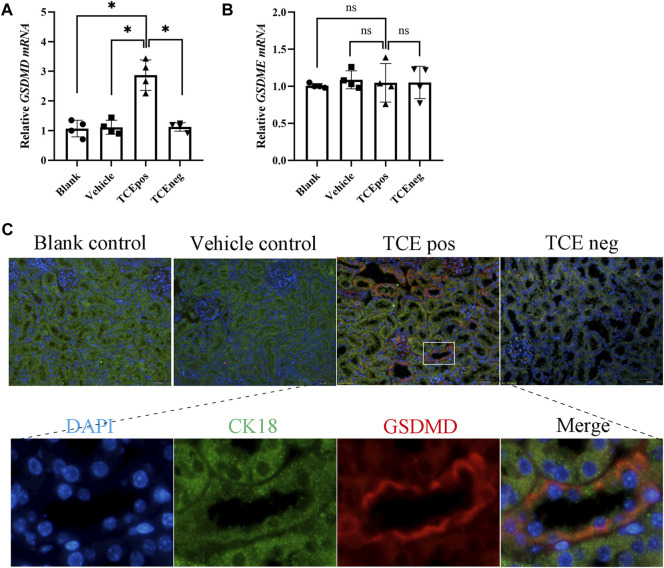
GSDMD-mediated RTEC pyroptosis contributed to TCE-induced renal damage. **(A,B)** Gene levels of executive proteins of pyroptosis, including GSDMD mRNA and GSDME mRNA. **(C)** Colocalization of GSDMD and CK18 (a hallmark of renal tubules) by dual immunofluorescence staining in each group. All the scale bars represent 50 μm. Data are representative of three independent experiments and are expressed as the mean ± SD. *n* = 3–5/group. Levels of significance were defined as below: *p < 0.05.

GSDMD mRNA was elevated in the TCEpos group when compared with the TCEneg group, while no difference in GSDMD mRNA was found among the blank control group, vehicle control group, and TCEneg group (*p* > 0.05). No statistical differences in GSDME were found among the abovementioned groups (*p* > 0.05) (Figures 3A,B). Next, protein expression of GSDMD was detected *via* immunofluorescence with the co-localization experiment. Using CK18 as the marker of RTECs, we found an overlap between GSDMD and CK18 in tubules from TCE-sensitized mice (Figure 3C).

### Caspase-1–Dependent Canonical Pathway Activated RTEC Pyroptosis

**FIGURE 4:**
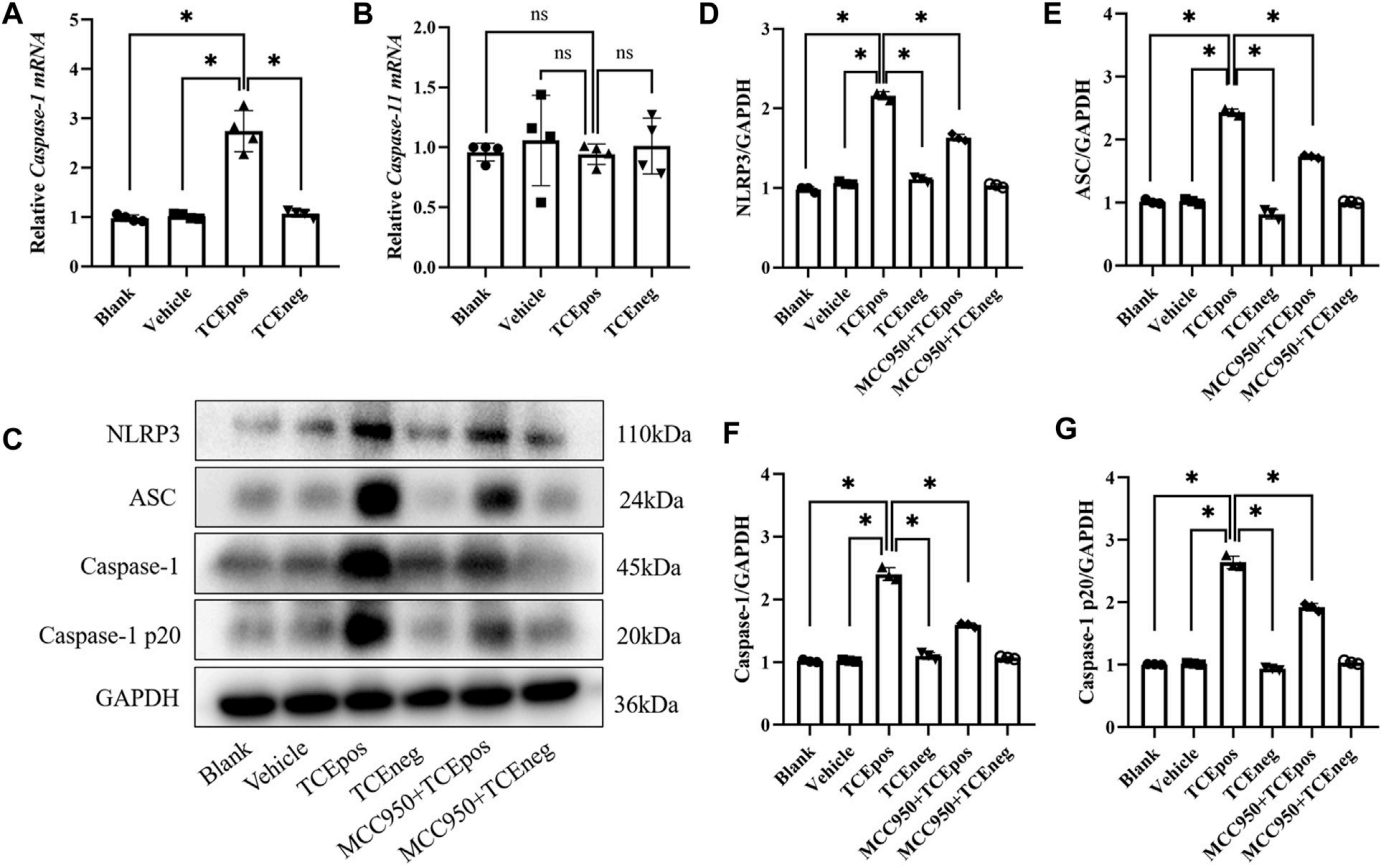
Caspase-1–dependent canonical pathway activated RTEC pyroptosis. **(A,B)** Gene levels of caspase-1 mRNA **(A)** and caspase-11 mRNA **(B)** in mice. **(C)** Representative immunoblot for expression of NLRP3, ASC, caspase-1, caspase-1 p20, and GAPDH. **(D–G)** Relative expression of the abovementioned proteins was assessed by the average optical density value in Image J. All the Western blot tests for NLRP3, ASC, caspase-1, and caspase-1 p20 were repeated three times, and the data are expressed as the mean ± SD. *n* = 3–5/group. Levels of significance were defined as below: *p < 0.05.

To gain more evidence of RTEC pyroptosis, we detected the expression of caspase-1 and caspase-11 which have been identified as the two major pathway proteins of GSDMD-mediated pyroptosis. An increased expression of caspase-1 mRNA, rather than caspase-11, was found in the TCEpos group (Figures 4A,B). Thus, we suspected that the caspase-1 pathway might be activated in RTEC-pyroptosis, and the key molecules of the caspase-1 pathway, including NLRP3, ASC, and caspase-1, were assessed by Western blotting. In line with RT-PCR, we showed that the expression of NLRP3 and ASC in the TCEpos group was higher than that of the TCEneg group and control groups (*p* < 0.05) (Figures 4C–E). In addition, both caspase-1 and caspase-1 p20 (active form of caspase-1) were upregulated in the TCEpos group compared with the TCEneg group or control groups (*p* < 0.05) (Figures 4C,F,G).

**FIGURE 5:**
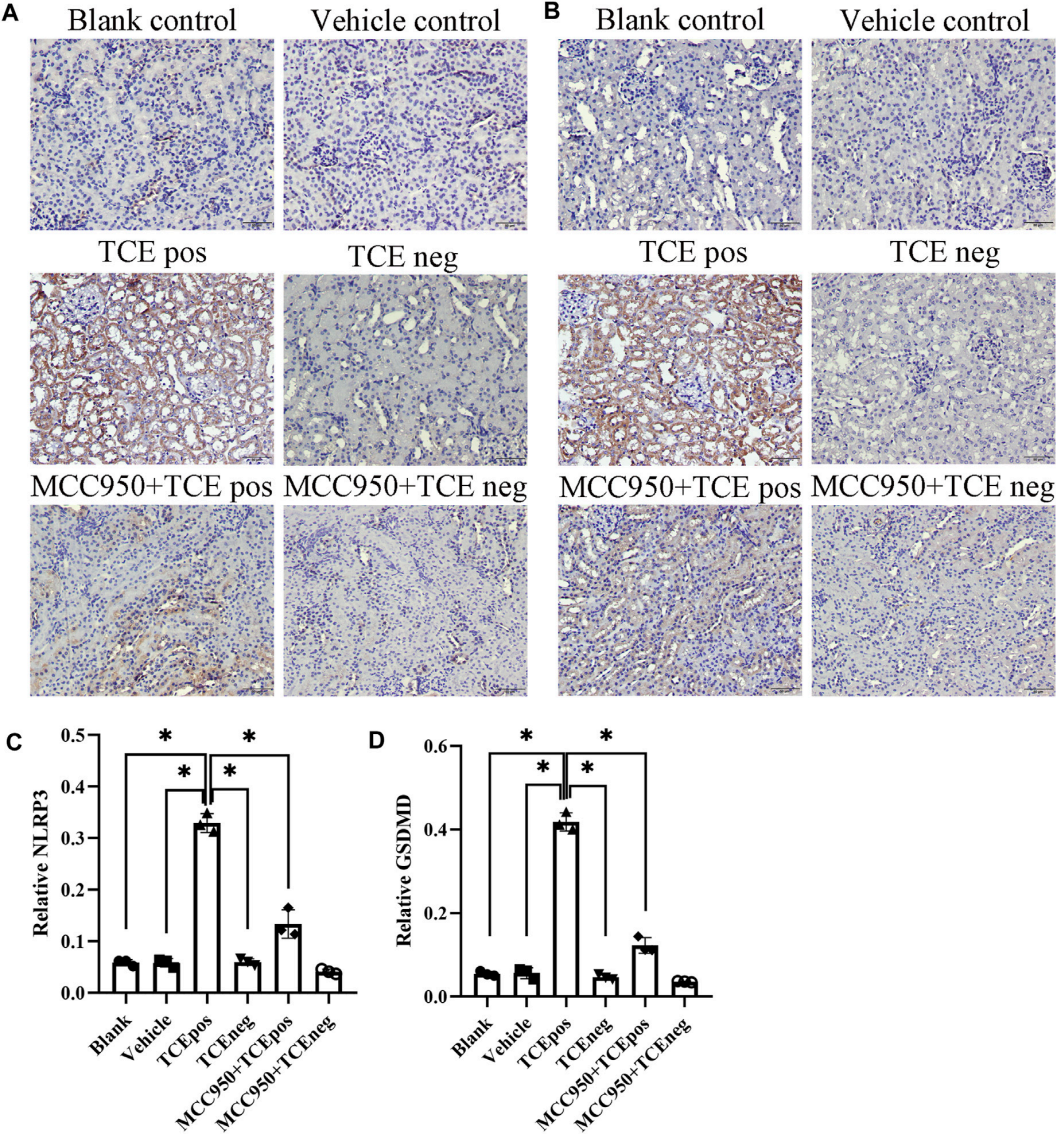
MCC950 pretreatment attenuated the expression of GSDMD in mouse kidney. Expression of NLRP3 **(A)** and GSDMD **(B)** in mouse kidney tissue. **(C,D)** Relative expression of NLRP3 **(C)** and GSDMD **(D)** was assessed by the average optical density value in Image J. All the scale bars represent 50 μm. Data are representative of three independent experiments and are expressed as the mean ± SD. *n* = 3–5/group. Levels of significance were defined as below: *p < 0.05.

**FIGURE 6:**
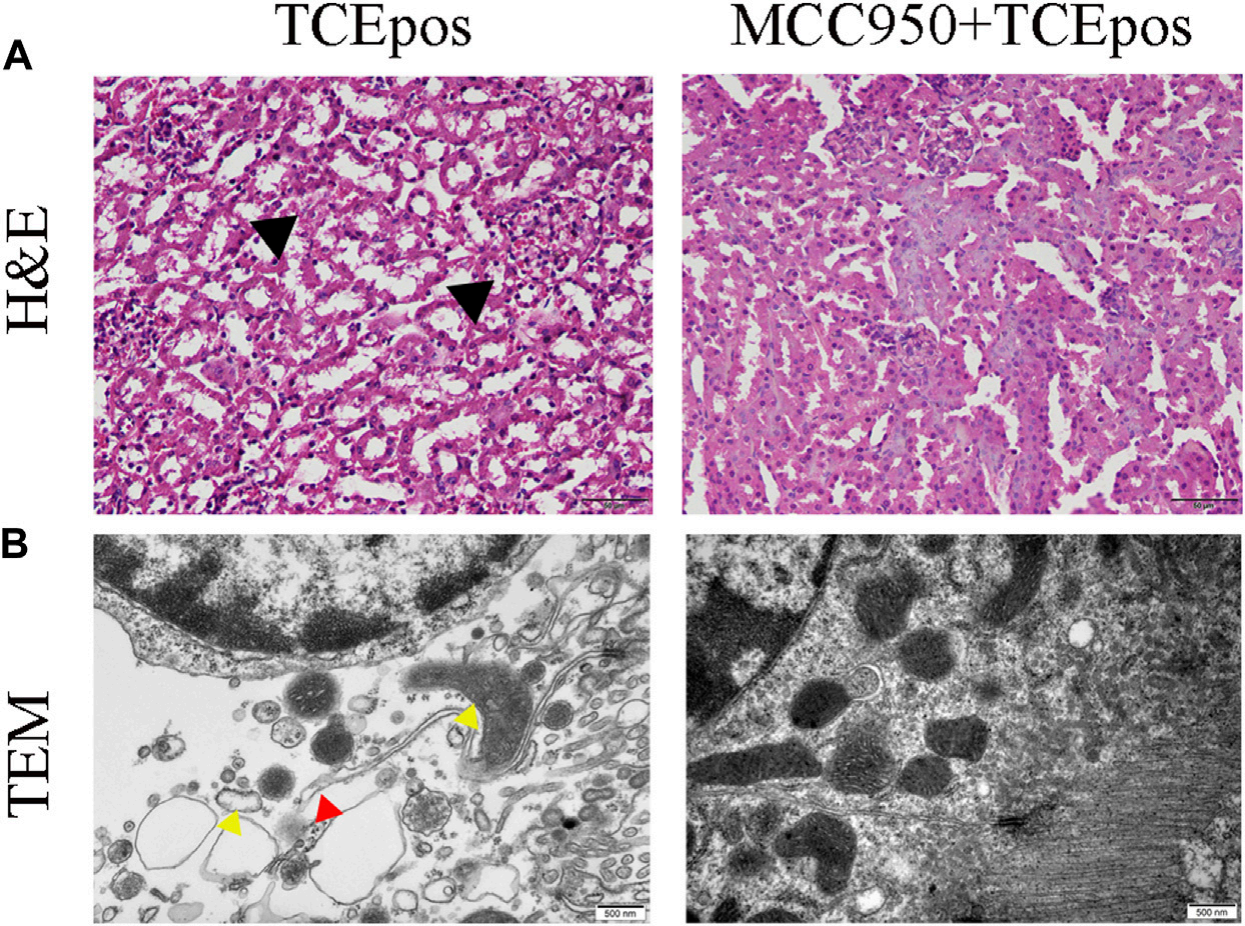
MCC950 pretreatment ameliorated the structural changes of renal tubules and RTECs in mice. **(A)** H&E staining of mouse kidney (magnification, ×400). The black arrow shows tubular swelling, vacuolar degeneration, or detachment of renal tubular epithelial cells. The scale bars represent 50 μm. **(B)** Transmission electron microscopy (TEM) of RTECs (magnification, ×30000). The red arrow shows the ruptured cytomembrane and the yellow arrow shows mitochondrial edema and vacuolar degeneration. The scale bars represent 500 nm. Data are representative images from three mice or independent experiments (*n* = 3).

Next, MCC950 was used to block the NLRP3/ASC/caspase-1 pathway and RTEC pyroptosis was examined. After caspase-1 pathway inhibition, the expression of NLRP3, ASC, caspase-1, and caspase-1 p20 was decreased in the MCC950 + TCEpos group compared with that in the TCEpos group (Figure 4C). Intriguingly, in addition to the NLRP3 inflammasome, the level of GSDMD was downregulated in the MCC950 + TCEpos group compared to that in the TCEpos group (Figure 5). Furthermore, we showed that MCC950 pretreatment could alleviate the changes of abnormal renal tubule structures and ultrastructural damage of RTECs, such as rupture cytomembrane, organelle reduction, mitochondrial edema, and vacuolar degeneration, in MCC905 + TCEpos mice (Figure 6). As expected, the pathological injury of renal tubules was relieved after caspase-1 pathway inhibition. Collectively, these findings suggested that the caspase-1 pathway was activated in RTEC pyroptosis, and pharmacological inhibition of this pathway might be protective against tubule injuries *via* restricting RTEC pyroptosis.

### C5b-9 Aggravated RTEC Damage in TCE-Sensitized Mice

**FIGURE 7:**
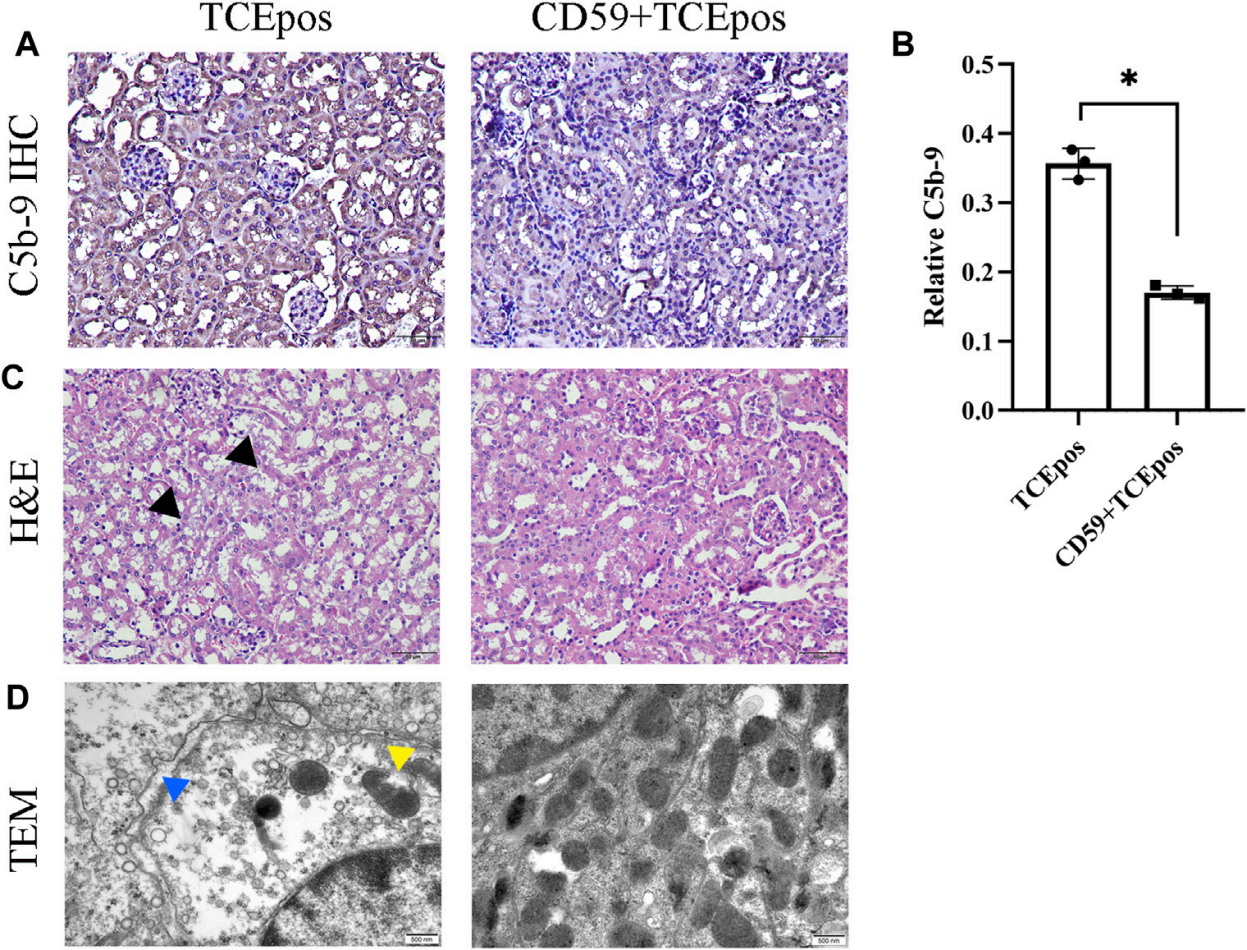
C5b-9 aggravated RTEC damage in TCE-sensitized mice. **(A)** Expression of C5b-9 in kidney tissue was analyzed by IHC. Twenty-five μg/mouse sCD59-cys pretreatment is effective against the assembly of C5b-9. Magnification, ×400. All the scale bars represent 50 μm. **(B)** Relative expression of C5b-9 assessed by the average optical density value in Image J. **(C)** H&E staining of the kidney (magnification, ×400). The black arrow shows tubular swelling, vacuolar degeneration, or detachment of renal tubular epithelial cells. The scale bars represent 50 μm. **(D)** Transmission electron microscopy of RTECs (magnification, ×30000). The yellow arrow shows mitochondrial edema and vacuolar degeneration, abnormal internal ridge structure, and ruptured mitochondrial membrane. The blue arrow shows an increased distance between cells. The scale bars represent 500 nm sCD59-cys pretreatment could ameliorate both the pathological changes of renal tubules and ultrastructural damage of RTECs. Data are representative of three independent experiments and are expressed as the mean ± SD. *n* = 3–5/group. Levels of significance were defined as below: *p < 0.05.

Consistent with our previous studies, we found that C5b-9 was deposited in tubules and sCD59-cys (25 μg/mouse) could effectively inhibit the assembly of C5b-9 (Figures 7A,B). To further determine the potential involvement of C5b-9 in RTEC injury, we examined the histopathological changes of renal tubules and ultrastructure of RTECs in the presence or absence of sCD59-cys pretreatment. A series of morphological changes, including obvious mitochondrial edema, vacuolar degeneration, abnormal internal ridge structure, a large reduction in organelles, and an increased distance between cells, were found in the TCEpos group, which were reduced to a large extent in the CD59 + TCEpos group (Figures 7C,D).

### C5b-9 Promoted GSDMD-Mediated RTEC Pyroptosis in TCE-Sensitized Mice

**FIGURE 8:**
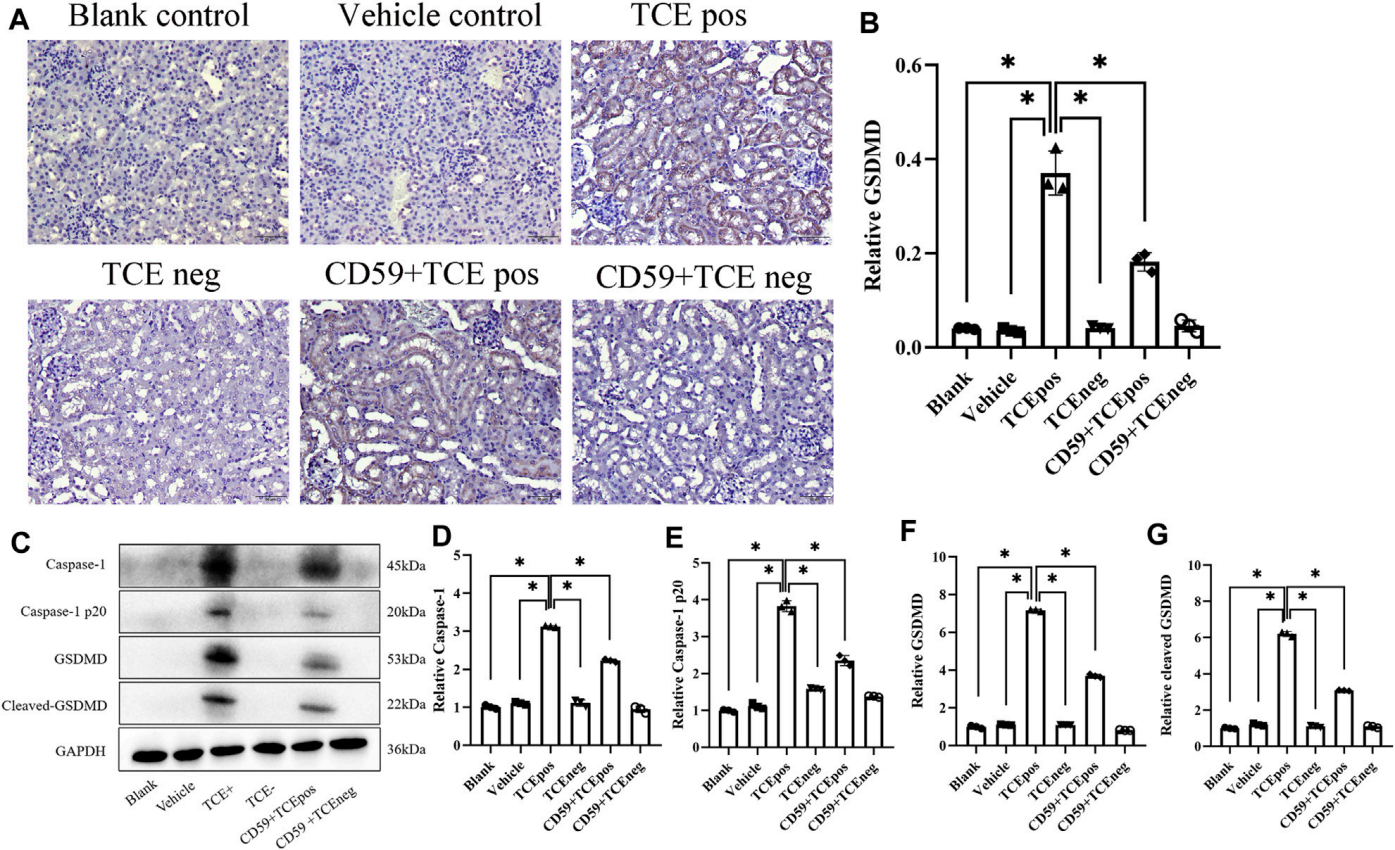
C5b-9 promoted GSDMD-mediated RTEC pyroptosis in TCE-sensitized mice. **(A)** Expression of GSDMD in mouse kidney by IHC. All the scale bars represent 50 μm *n* = 5/group. **(B)** Relative expression of GSDMD in kidney tissue assessed by average optical density value in Image J. **(C)** Representative immunoblot for expression of caspase-1, caspase-1 p20, GSDMD, and cleaved-GSDMD. **(D–G)** Relative expression of caspase-1, caspase-1 p20, GSDMD, and cleaved-GSDMD by optical density values in Image J. All the Western blot tests for the abovementioned proteins were repeated three times. *n* = 3–5/group. The data are expressed as the mean ± SD. Levels of significance were defined as below: *p < 0.05.

To further investigate whether C5b-9 was involved in the pyroptosis of RTECs in TCE-sensitized mice, we examined the activation of the RTEC pyroptosis executor GSDMD and caspase-1–dependent pathway with or without intraperitoneal injection of the recombinant protein CD59 (C5b-9 inhibitor). The expression of GSDMD in mouse renal cortex was detected by IHC, and a decrease of GSDMD in the CD59 + TCEpos group than the TCEpos group was observed (*p* < 0.05) (Figures 8A,B). In line with IHC, Western blotting data showed that both levels of caspase-1 and caspase-1 p20 were decreased in the CD59 + TCEpos group compared with the TCEpos group (*p* < 0.05). Similarly, the expression of GSDMD and cleaved GSDMD in the CD59 + TCEpos group was lower than that in the TCEpos group (*p* < 0.05) (Figures 8C–G).

## Discussion

OMDT is a rare but life-threatening occupational health problem (Li et al., 2019). Unlike general chemical-induced irritant contact dermatitis, OMDT is often accompanied with severe inflammatory visceral complications, including hepatitis and immune kidney damage (Wang et al., 2021). Although not as fatal as liver damage, immune kidney damage is proved to be closely related to disease severity and prognosis (Liu, 2009). In OMDT patients, the immune kidney injuries mainly presented as diffuse inflammation, positive urine protein, and renal dysfunction with abnormal expression of ALB, α1-MG, and β2-MG (Liu, 2009; Wen et al., 2019). In addition, an altered level of inflammation-related cytokines such as TNF-α, IL-6, and IL-10 was also found in the serum of OMDT patients (Xueqin et al., 2018). In accordance with human reports, the present study and our previous studies showed that renal tubular inflammatory injuries were present in TCE-induced kidney damage, as evidenced by renal pathological examination and overexpression of IL-1β and IL-18 in renal tubules (Wang Y. et al., 2020; Xie et al., 2021).

Pyroptosis is a unique type of programed cell death depending on inflammatory caspase–mediated activation of GSDMD (Li et al., 2021). Activated GSDMD protein formed the pore in the cell membrane, causing cellular swell and rupture and the release of proinflammatory IL-1β and IL-18 (Ding et al., 2021). Recent studies have indicated that pyroptosis could amplify the local inflammatory response in kidney disorders, including diabetic kidney disease and acute kidney injury (Shahzad et al., 2015; Tan et al., 2017). Wang et al. suggested that GSDMD-mediated pyroptosis contributes to the pathogenesis of LPS-induced acute liver failure and concomitant acute kidney injury with mechanisms involving TNF-a/HMGB-1 signaling (Wang G. et al., 2020). Naijun Miao et al. also demonstrated that the key role of RTEC pyroptosis in acute kidney injury was associated with IL-18 excretion in urine (Miao et al., 2019). To investigate whether pyroptosis is involved in TCE-induced kidney damage, we tested the two main executive molecules, GSDMD and GSDME. We found an increase of *GSDMD* mRNA in TCE-sensitized mice, while non-obvious changes of GSDME mRNA occurred in other groups, and GSDMD protein was expressed in renal tubules and located in the RTECs. Thus, we revealed a new mechanism of RTEC pyroptosis in TCE-induced kidney damage which, to our knowledge, has never been reported in literature.

Studies have shown that caspase-1 activation is initiated by the assembly of the NLRP3 inflammasome, comprised of NLRP3, ASC, and caspase-1, and activated caspase-1 promoted the secretion of mature IL-1β and IL-18 to amplify the inflammatory response (Li et al., 2021; Sun et al., 2022). We previously reported the increase of NLRP3 signaling in TCE-induced kidney damage (Xie et al., 2021). Here, we isolated primary RTECs to test the activation of NLRP3 signaling at the cellular level. Our results showed that the NLRP3 signaling contributed to TCE-induced RTEC damage, and after pharmacological inhibition of the NLRP3/caspase-1 pathway, the activation of downstream GSDMD was effectively reduced associated with obvious reversal of ultrastructural changes found in murine RTECs. Therefore, we speculated that the activation of caspase-1–dependent canonical pathway functions to trigger the GSDMD-mediated RTEC pyroptosis in TCE-sensitized mice.

Our previous study found that C5b-9 mainly played a sub-lytic role in the process of renal tubular injury in TCE-sensitized mice (Wang X. et al., 2019). Indeed, as a membrane pore–forming protein, the sub-lytic effects of C5b-9 play a more important role in inflammatory response (Zhang et al., 2021). Studies have shown that sub-lytic C5b-9 worked as a trigger of various intracellular signaling processes such as MAPK (Zhu et al., 2017), NF-κB (Guo et al., 2020), and the NLRP3 (Zhang et al., 2021) signaling. However, whether sub-lytic C5b-9 is involved in TCE-induced RTEC pyroptosis remains unknown. In the current study, we pretreated mice with recombinant CD59 to block the deposition of C5b-9 on RTECs. In line with our previous study, we found that C5b-9 exerted sub-lytic effects in tubular damage. In addition, our data supported that the sub-lytic C5b-9 promotes the RTEC pyroptosis in TCE-sensitized mice based on the observed downregulation of caspase-1 and GSDMD after CD59 pretreatment.

Therefore, we provided a novel insight on C5b-9 in mediating immune response in TCE-induced kidney damage. A recent *in vitro* study uncovered that sub-lytic C5b-9 could directly activate NLRP3 via internalization into endosomes and colocalized with the components of NLRP3 (Diaz-Del-Olmo et al., 2021). With the function of NLRP3, caspase-1 was activated and then cleaved full-length GSDMD, generating an active form of GSDMD and mediating proinflammatory response (Diaz-Del-Olmo et al., 2021). In addition, another study suggested that the deposition of C5b-9 on the cytomembrane opened a pore for the influx of calcium, which is an essential signal for NLRP3 activation (Kumar et al., 2018). Unfortunately, due to the lack of *in vitro* models for visceral injury by TCE sensitization, we could not further explore the precise mechanism as to how C5b-9 works on NLRP3 and caspase-1–mediated pyroptosis in the absence of the immune environment of TCE sensitization. We will be sought to resolve this problem in our future studies.

## Conclusion

In conclusion, we provide new evidence that RTEC pyroptosis contributes to diffuse kidney inflammatory damage. The caspase-1–dependent canonical pathway was activated in TCE-induced renal tubular damage. Pharmacological inhibition of C5b-9 could restrain the RTEC pyroptosis and rescue renal tubular damage. Collectively, our finding suggests a novel mechanism of NLRP3-mediated RTEC pyroptosis in TCE-induced kidney damage. Drugs targeting C5b-9 and pyroptosis might be a new choice for clinical treatment of kidney damage in TCE sensitization.

## Data Availability

The original contributions presented in the study are included in the article/Supplementary Material, further inquiries can be directed to the corresponding author.

## References

[B1] Barnett K. C. Ting J. P. (2020). Mitochondrial GSDMD Pores DAMPen Pyroptosis. Immunity 52 (3), 424–426. 10.1016/j.immuni.2020.02.012 32187511 PMC7337261

[B2] Broz P. Pelegrín P. Shao F. (2020). The Gasdermins, a Protein Family Executing Cell Death and Inflammation. Nat. Rev. Immunol. 20 (3), 143–157. 10.1038/s41577-019-0228-2 31690840

[B3] Cheng K. T. Xiong S. Ye Z. Hong Z. Di A. Tsang K. M. (2017). Caspase-11-mediated Endothelial Pyroptosis Underlies Endotoxemia-Induced Lung Injury. J. Clin. Invest. 127 (11), 4124–4135. 10.1172/JCI94495 28990935 PMC5663346

[B4] Diaz-Del-Olmo I. Worboys J. Martin-Sanchez F. Gritsenko A. Ambrose A. R. Tannahill G. M. (2021). Internalization of the Membrane Attack Complex Triggers NLRP3 Inflammasome Activation and IL-1β Secretion in Human Macrophages. Front. Immunol. 12, 720655. 10.3389/fimmu.2021.720655 34650553 PMC8506164

[B5] Ding J. Wang K. Liu W. She Y. Sun Q. Shi J. (2016). Pore-forming Activity and Structural Autoinhibition of the Gasdermin Family. Nature 535 (7610), 111–116. 10.1038/nature18590 27281216

[B6] Ding X. Kambara H. Guo R. Kanneganti A. Acosta-Zaldívar M. Li J. (2021). Inflammasome-mediated GSDMD Activation Facilitates Escape of Candida Albicans from Macrophages. Nat. Commun. 12 (1), 6699. 10.1038/s41467-021-27034-9 34795266 PMC8602704

[B7] Elkin E. R. Bakulski K. M. Colacino J. A. Bridges D. Kilburn B. A. Armant D. R. (2021). Transcriptional Profiling of the Response to the Trichloroethylene Metabolite S-(1,2-dichlorovinyl)-L-cysteine Revealed Activation of the eIF2α/ATF4 Integrated Stress Response in Two *In Vitro* Placental Models. Arch. Toxicol. 95 (5), 1595–1619. 10.1007/s00204-021-03011-5 33725128 PMC7961173

[B8] Guo J. Li M. Yang Y. Zhang L. Zhang L. W. Sun Q. Y. (2020). Pretreatment with Atorvastatin Ameliorates Cobra Venom Factor-Induced Acute Lung Inflammation in Mice. BMC Pulm. Med. 20 (1), 263. 10.1186/s12890-020-01307-3 33046059 PMC7552367

[B9] Harris A. P. Ismail K. A. Nunez M. Martopullo I. Lencinas A. Selmin O. I. (2018). Trichloroethylene Perturbs HNF4a Expression and Activity in the Developing Chick Heart. Toxicol. Lett. 285, 113–120. 10.1016/j.toxlet.2017.12.027 29306027 PMC5803365

[B10] Kayagaki N. Stowe I. B. Lee B. L. O'Rourke K. Anderson K. Warming S. (2015). Caspase-11 Cleaves Gasdermin D for Non-canonical Inflammasome Signalling. Nature 526 (7575), 666–671. 10.1038/nature15541 26375259

[B11] Kumar B. Cashman S. M. Kumar-Singh R. (2018). Complement-Mediated Activation of the NLRP3 Inflammasome and its Inhibition by AAV-Mediated Delivery of CD59 in a Model of Uveitis. Mol. Ther. 26 (6), 1568–1580. 10.1016/j.ymthe.2018.03.012 29678656 PMC5986727

[B12] Li S. Sun Y. Song M. Song Y. Fang Y. Zhang Q. (2021). NLRP3/caspase-1/GSDMD-mediated Pyroptosis Exerts a Crucial Role in Astrocyte Pathological Injury in Mouse Model of Depression. JCI Insight 6 (23). 10.1172/jci.insight.146852 PMC8675200 34877938

[B13] Li W. Liu X. Yang X. Chen Y. Pang Y. Qi G. (2019). Effect of Trichloroacetaldehyde on the Activation of CD4+T Cells in Occupational Medicamentosa-like Dermatitis: An *In Vivo* and *In Vitro* Study. Toxicology 423, 95–104. 10.1016/j.tox.2019.05.014 31150805

[B14] Liu J. (2009). Clinical Analysis of Seven Cases of Trichloroethylene Medicamentose-like Dermatitis. Ind. Health 47 (6), 685–688. 10.2486/indhealth.47.685 19996547

[B15] Miao N. Yin F. Xie H. Wang Y. Xu Y. Shen Y. (2019). The Cleavage of Gasdermin D by Caspase-11 Promotes Tubular Epithelial Cell Pyroptosis and Urinary IL-18 Excretion in Acute Kidney Injury. Kidney Int. 96 (5), 1105–1120. 10.1016/j.kint.2019.04.035 31405732

[B16] Morgan B. P. Walters D. Serna M. Bubeck D. (2016). Terminal Complexes of the Complement System: New Structural Insights and Their Relevance to Function. Immunol. Rev. 274 (1), 141–151. 10.1111/imr.12461 27782334

[B17] Ordaz J. D. Damayanti N. P. Irudayaraj J. M. K. (2017a). Toxicological Effects of Trichloroethylene Exposure on Immune Disorders. Immunopharmacol Immunotoxicol 39 (6), 305–317. 10.1080/08923973.2017.1364262 28828896

[B18] Ordaz J. D. Damayanti N. P. Irudayaraj J. M. K. (2017b). Toxicological Effects of Trichloroethylene Exposure on Immune Disorders. Immunopharmacol Immunotoxicol 39 (6), 305–317. 10.1080/08923973.2017.1364262 28828896

[B19] Serna M. Giles J. L. Morgan B. P. Bubeck D. (2016). Structural Basis of Complement Membrane Attack Complex Formation. Nat. Commun. 7, 10587. 10.1038/ncomms10587 26841837 PMC4743022

[B20] Shahzad K. Bock F. Dong W. Wang H. Kopf S. Kohli S. (2015). Nlrp3-inflammasome Activation in Non-myeloid-derived Cells Aggravates Diabetic Nephropathy. Kidney Int. 87 (1), 74–84. 10.1038/ki.2014.271 25075770 PMC4284813

[B21] Sun X. Liu Y. Huang Z. Xu W. Hu W. Yi L. (2022). SARS-CoV-2 Non-structural Protein 6 Triggers NLRP3-dependent Pyroptosis by Targeting ATP6AP1. Cell Death Differ. 10.1038/s41418-021-00916-7 PMC9177730 34997207

[B22] Tan X. Zheng X. Huang Z. Lin J. Xie C. Lin Y. (2017). Involvement of S100A8/A9-TLR4-NLRP3 Inflammasome Pathway in Contrast-Induced Acute Kidney Injury. Cell. Physiol. Biochem. 43 (1), 209–222. 10.1159/000480340 28854431

[B23] Wang F. Dai Y. Huang M. Zhang C. Huang L. Wang H. (2021). Glomerular Damage in Trichloroethylene-Sensitized Mice: Targeting Cathepsin L-Induced Hyperactive mTOR Signaling. Front. Pharmacol. 12, 639878. 10.3389/fphar.2021.639878 34393767 PMC8358928

[B24] Wang F. Huang L. P. Dai Y. Y. Huang M. Jiang W. Ye L. P. (2019a). Terminal Complement Complex C5b-9 Reduced Megalin and Cubilin-Mediated Tubule Proteins Uptake in a Mouse Model of Trichloroethylene Hypersensitivity Syndrome. Toxicol. Lett. 317, 110–119. 10.1016/j.toxlet.2019.10.002 31618666

[B25] Wang G. Zhang J. Dai Y. Xu Q. Zhu Q. (2020a). Local Renal Complement Activation Mediates Immune Kidney Injury by Inducing Endothelin-1 Signalling and Inflammation in Trichloroethylene-Sensitised Mice. Toxicol. Lett. 333, 130–139. 10.1016/j.toxlet.2020.07.036 32763311

[B26] Wang H. Zhang J. X. Li S. L. Wang F. Zha W. S. Shen T. (2015). An Animal Model of Trichloroethylene-Induced Skin Sensitization in BALB/c Mice. Int. J. Toxicol. 34 (5), 442–453. 10.1177/1091581815591222 26111540

[B27] Wang X. Yu Y. Xie H. B. Shen T. Zhu Q. X. (2019b). Complement Regulatory Protein CD59a Plays a Protective Role in Immune Liver Injury of Trichloroethylene-Sensitized BALB/c Mice. Ecotoxicol Environ. Saf. 172, 105–113. 10.1016/j.ecoenv.2019.01.049 30685621

[B28] Wang Y. Zhang H. Chen Q. Jiao F. Shi C. Pei M. (2020b). TNF-α/HMGB1 Inflammation Signalling Pathway Regulates Pyroptosis during Liver Failure and Acute Kidney Injury. Cell Prolif 53 (6), e12829. 10.1111/cpr.12829 32419317 PMC7309595

[B29] Wen L. Zhao Z. Wang Z. Xiao J. Birn H. Gregersen J. W. (2019). High Levels of Urinary Complement Proteins Are Associated with Chronic Renal Damage and Proximal Tubule Dysfunction in Immunoglobulin A Nephropathy. Nephrology (Carlton) 24 (7), 703–710. 10.1111/nep.13477 30141239

[B30] Xie H. Yang L. Yang Y. Jiang W. Wang X. Huang M. (2021). C5b-9 Membrane Attack Complex Activated NLRP3 Inflammasome Mediates Renal Tubular Immune Injury in Trichloroethylene Sensitized Mice. Ecotoxicol Environ. Saf. 208, 111439. 10.1016/j.ecoenv.2020.111439 33039874

[B31] Xueqin Y. Wenxue L. Peimao L. Wen Z. Xianqing H. Zhixiong Z. (2018). Cytokine Expression and Cytokine-Based T-Cell Profiling in Occupational Medicamentosa-like Dermatitis Due to Trichloroethylene. Toxicol. Lett. 288, 129–135. 10.1016/j.toxlet.2018.02.012 29477354

[B32] Zhang C. Gong Y. Li N. Liu X. Zhang Y. Ye F. (2021). Long Noncoding RNA Kcnq1ot1 Promotes sC5b-9-Induced Podocyte Pyroptosis by Inhibiting miR-486a-3p and Upregulating NLRP3. Am. J. Physiol. Cel Physiol 320 (3), C355–C364. 10.1152/ajpcell.00403.2020 33296289

[B33] Zhu G. Qiu W. Li Y. Zhao C. He F. Zhou M. (2017). Sublytic C5b-9 Induces Glomerular Mesangial Cell Apoptosis through the Cascade Pathway of MEKK2-P38 MAPK-IRF-1-TRADD-Caspase 8 in Rat Thy-1 Nephritis. J. Immunol. 198 (3), 1104–1118. 10.4049/jimmunol.1600403 28039298

