# Supplementary material for: Membrane Attack Complex C5b-9 Promotes Renal Tubular Epithelial Cell Pyroptosis in Trichloroethylene-Sensitized Mice
Source: Front Pharmacol. 2022 May 17;13:877988. doi: 10.3389/fphar.2022.877988 (PMC9152256; doi:10.3389/fphar.2022.877988)

**Supplementary figures of whole uncropped images of the original western blots and original microscopy images**

**All original microscopy images:**

**https://www.jianguoyun.com/p/DaXy1IsQ57ejChip-q0E**

**The whole uncropped images of FIGURE 4C：**

ASC with ladder:





Caspase-1+Caspase-1 p20 with ladder:





NLRP3 with ladder:


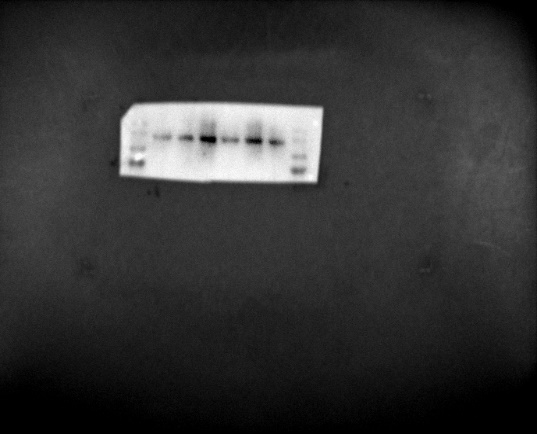


GAPDH with ladder:


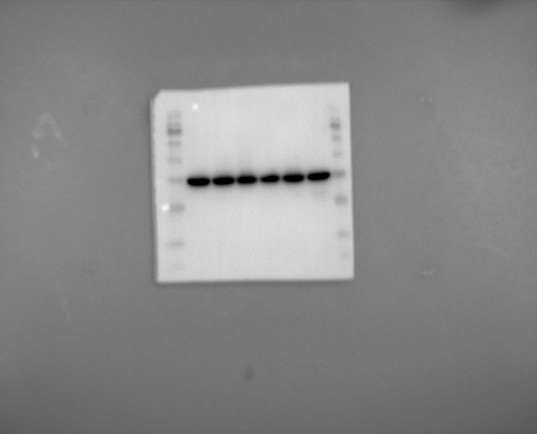


**The whole uncropped images of FIGURE 8C：**

Caspase-1+Caspase-1 p20 with ladder:


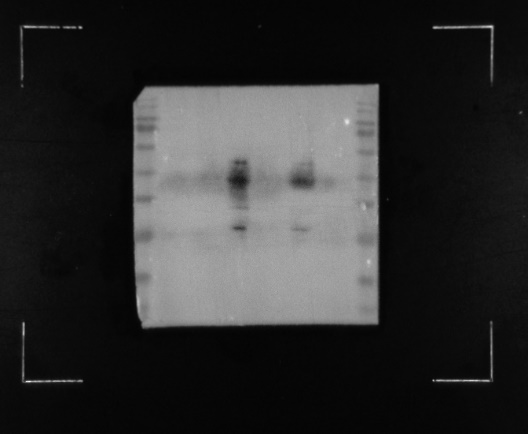


GSDMD+Cleaved-GSDMD with ladder:


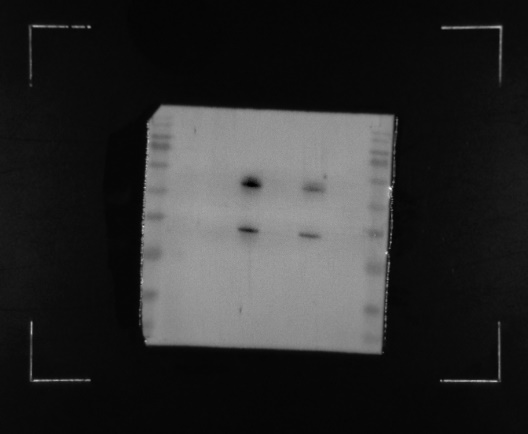


GAPDH with ladder：


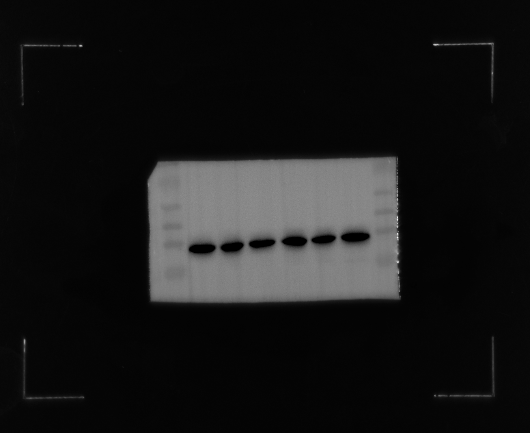

Supplement: Supplementary file 1 [file DataSheet1.DOCX]
